# Prevalence and Associated Risk Factors of Endoparasites among Under-Five Children in Debre Tabor Comprehensive Specialized Hospital, Debre Tabor, Northwest Ethiopia: A Cross-Sectional Study

**DOI:** 10.1155/2022/6917355

**Published:** 2022-04-30

**Authors:** Atalel Eyasu, Mulugeta Molla, Belayneh Kefale, Woretaw Sisay, Yared Andargie, Fassikaw Kebede, Tadeg Jemere

**Affiliations:** ^1^Agissa Health Center, Mena Meketewa, Ethiopia; ^2^Pharmacology and Toxicology Unit, Department of Pharmacy, College of Health Sciences, Debre Tabor University, Debre Tabor, Ethiopia; ^3^Clinical Pharmacy Unit, Department of Pharmacy, College of Medical and Health Sciences, Bahir Dar University, Bahir Dar, Ethiopia; ^4^Department of Epidemiology and Biostatistics, School of Public Health, College of Health Science, Woldia University, Woldia, Ethiopia; ^5^Department of Biomedical Sciences, College of Health Sciences, Debre Tabor University, Debre Tabor, Ethiopia

## Abstract

Many endoparasites are still considered neglected tropical illnesses. The term “endoparasites” refers to infections caused by both helminths and protozoa. In many places in Ethiopia, particularly Debre Tabor, epidemiological data on the prevalence and associated variables of endoparasites among under-five children is unavailable. Thus, the aim of this study was to gather baseline data on the prevalence of endoparasites and their associated variables among under-five children who visited the Debre Tabor comprehensive specialized hospital in Northwest Ethiopia. A hospital-based quantitative cross-sectional study was used. The study was carried out from May 1 to November 30, 2021. Study participants were selected by a systematic sampling technique. The stool specimen was examined for the presence of different stages of intestinal parasites (adult, trophozoite, larvae, cysts, and ova) using direct wet mount, modified formal-ether sedimentation, and modified Ziehl–Neelsen methods. The IBM SPSS statistical package (version 23) was used to enter and analyze the collected data. The data was summarized using frequency tables and a bar chart. The adjusted odds ratio and *p* value *<*0.05 were used to declare the final association. In the present study, a total of 258 under-five children and their mothers/guardians were involved in the study, with a response rate of 100%. More than half of the respondents, 137 (53.10%), were females, and 159 (61.63%) were in the age group of 24 to 59 months. The overall prevalence of one or more endoparasites among under-five children was 45 (17.44%). Multivariate logistic regression analysis showed that health supervision, child food freshness, regular trimming of fingernails, and children's playground cleanliness were significantly associated with childhood endoparasites. The present study demonstrated a higher prevalence of endoparasites among under-five children. Health supervision, child food freshness, regular trimming of fingernails, and children's playground cleanliness were significantly associated with endoparasites. Thus, strengthening health education about food, personal, and environmental hygiene for both children and their mothers/guardians is crucial.

## 1. Introduction

Many endoparasites are still considered neglected tropical illnesses. The term “endoparasites” refers to infections caused by both helminths and protozoa [1]. The symptoms of endoparasites include anemia, asthma, weight loss, fatigue, low immune system, nervousness, skin rash, diarrhea, vomiting, loss of appetite, digestive disorders, abdominal discomfort, and an enlarged abdomen [2, 3]. Children's physical and mental growth and development are harmed by chronic endoparasites in general. In addition, endoparasites may increase susceptibility to infections with other intestinal pathogens [4].

According to the World Health Organization, more than 270 million preschoolers and 600 million schoolchildren live in locations where parasites are widely spread [5]. Studies have shown that endoparasites are a serious childhood health problem in several developing countries, including Ethiopia, where the problem is exacerbated by a poor environmental and personal hygiene, a lack of health knowledge, and poor socioeconomic situations [2, 6, 7]. Two-thirds (2/3) of African countries had high-risk areas with a prevalence of more than 50% [8].

In Ethiopia, the prevalence of endoparasites is significant. The overall national prevalence of any helminth infection was 29.8%, with regional incidence varying significantly [9]. In previous studies in Ethiopian cities and rural regions, the prevalence of endoparasites among under-five children ranged from 15.5% to 85.1% [2, 5, 6, 10–21]. *Ascaris lumbricoides* (10.77%), *Giardia lamblia* (10.45%), and hookworm (7.88%) were found to be more common in the Boricha district of South Ethiopia [20]. The situation is worsening in the region, as indicated by a study published in Wondo Genet that revealed a high incidence of *Trichuris trichiura* (74.7%) and *Schistosoma mansoni* (37.2%) [2]. *Hymenolepis nana* prevalence was found to be 21.4% in Senbete and Bete towns [6].

Previous study has found a relationship between endoparasites and socioeconomic characteristics, with low maternal education, low family income, residing in a rural region, and poor sanitary facilities all being associated with a higher prevalence of endoparasites [22]. People become infected by ingesting infective stages of the parasites (eggs and cysts) or being bitten by the larvae stage of the parasites through contaminated soil, water, and undercooked meat and/or vegetables [23, 24].

Endoparasites are frequent in under-five children and can be caused by a variety of factors, such as playing in the dirt, sucking fingers, or defecating in an open field. The prevalence of endoparasites is influenced by maternal understanding of their prevention and control. Regular antihelminthic therapy, increased access to potable water, sanitation, and health education are all important to limit the effects of endoparasites [3, 10, 25].

In many places in Ethiopia, particularly Debre Tabor, epidemiological data on the prevalence and associated variables of endoparasites among under-five children is unavailable. Because their immune systems have not fully developed, under-five children require extra attention and follow-up because they are more prone to heavy infections with endoparasites and other infectious diseases, and they frequently play in fecal-contaminated dirt [26, 27]. Thus, the aim of this study was to gather baseline data on the prevalence of endoparasites and their associated variables among under-five children who visited the Debre Tabor comprehensive specialized hospital in Northwest Ethiopia. This information could help public health planners, policymakers, and implementers in developing and implementing effective intervention strategies to reduce related morbidity and mortality among under-five children.

## 2. Methods and Materials

### 2.1. Study Area

This study was conducted in the Debre Tabor comprehensive specialized hospital's pediatric ward. It is located 667 kilometers Northwest of Ethiopia's capital, Addis Ababa, in Debre Tabor, a town in the South Gondar zone. The town is located at latitude 11°51°E -38°1°E and longitude 11.850°N–38.017°E. Above sea level, the elevation is 2706 meters (8878 feet). The overall population of the South Gondar zone, according to the Federal Democratic Republic of Ethiopia National Statistics Bureau Population Projections for 2014–2017 reports, was 2,484,929, with 1,257,323 men and 1,227,606 women [28]. There are 405 health posts, 96 health facilities, eight basic hospitals, and one comprehensive specialized hospital located in the South Gondar zone. This hospital serves a total population of 2.3 million people. Four town administrations and 14 districts are included in the hospital's catchment region. There are 120 beds and six admittance wards (pediatric, medical, surgical, gynecological, obstetrics, neonatal, and psychiatric) in the facility [29].

### 2.2. Study Design and Period

A hospital-based quantitative cross-sectional study was used. The study was carried out from May 1 to November 30, 2021.

### 2.3. Inclusion Criteria

Under-five children who had sufficient stool and were not on an antiparasitic drug within one month before screening were included. Children who were critically ill and mothers/guardians who did not agree to give information about their children were excluded from the study.

### 2.4. Sample Size Determination and Sampling Procedure

The sample size (*n*) for the prevalence of endoparasites was calculated using the single population proportion formula with an endoparasites rate of 18.7% (*P*) [12] at Woreta Health Center, a sampling error of 5% (*d*), and a 95% confidence interval (*Zα*/2). Based on the assumption of a 10% nonresponse rate, the final sample size was 258. The sample size for factors associated with endoparasites was calculated using OpenEpi version 3 and taking into account the study's 80% power, 95% two-sided confidence interval, 1 : 1 case to control ratio, and 10% nonresponse rate. To determine sample size, the least odds ratio and proportion of cases with exposure to factors linked to endoparasites in prior studies were chosen. As a result, sample size was determined by accounting for independent variables such as age, education, and occupation [11, 16]. Finally, the survey's largest sample size (258) was considered. Study participants were selected by a systematic sampling technique; every fourth child was included based on the arrival order of their hospital visit.

### 2.5. Data Collection Tool

Data was collected using a structured interview questionnaire, which was prepared after an intensive review of related literature on the topic [2, 5, 6, 10–21]. The tool contained two parts. The first part embraced questions about the sociodemographic profile of respondents, such as gender and age of child, family residence, religion, ethnicity, occupation, mother/guardian's educational status, and family income. The second part was categorized into two subheadings. The first was to inquire about health information. The second was characteristics of food, personal, and environmental hygiene. The data collection tool was initially prepared in an English version, which was later translated into an Amharic version, the local language, and then retranslated to English for analysis to ensure consistency (Supplementary material 1). The content validity was determined by a team of specialists from the fields of public health, medical laboratory science, epidemiology, and biostatistics. The final version of the questionnaire contained a reliable indicator, which was a good sign (as indicated by the Alpha Cronbach test value of 0.902).

### 2.6. Data Quality Control and the Data Collection Processes

One week prior to the actual data collection period, the questionnaire was pretested on 13 under-five children served in Ebinat Primary Hospital, Northwest Ethiopia, to ensure its appropriateness. It was corrected and used after the pretest. The collected questionnaires were reviewed on a daily basis for completeness, accuracy, clarity, and consistency of data. Data was collected by four diploma nurses and two laboratory technologists by face-to-face interview in the local language (Amharic) using a structured questionnaire guide.

The stool specimen was examined for the presence of different stages of intestinal parasites (adult, trophozoite, larvae, cysts, and ova) using direct wet mount, modified formal-ether sedimentation, and modified Ziehl–Neelsen methods. A total of 258 fresh stool samples were collected in labeled vials and were processed within one hour of collection at the clinical parasitology laboratory, Debre Tabor comprehensive specialized hospital.

In the direct wet mount method, direct normal saline (0.85% NaCl solution) and Lugol's iodine wet mount of each sample were used to detect endoparasites microscopically. The wet mounts were examined under a light microscope (CX21FS1, Olympus Corporation, Philippines) at 100× and 400× magnifications. A small portion of the stool specimen was also preserved in 10% formalin for repeating the tests whenever required and for further analysis [20].

In the modified formal-ether sedimentation method, a portion of each preserved stool specimen was taken and processed. Briefly, 1 g of stool was placed in a clean conical centrifuge tube containing 7 mL of 10% formol water by using an applicator stick and shacked gently. The resulting suspension was filtered through a sieve into another conical tube. After adding 3–4 mL of diethyl ether to the formalin solution, the content was centrifuged at 3000 revolutions per minute for 1 minute. The supernatant was discarded, and the tube was reinstalled in its rack. Finally, a smear was prepared from the sediment and observed under a light microscope with a magnification of 100× and 400× [20].

The entire negative specimens and 10% of the total positive slides were randomly selected and reexamined by another blinded technician. To ensure the quality of the investigation, the two readers independently read the slides, and their readings were compared. Discordants were immediately resolved with a discussion of each other and in consultation with other experts. To ensure the validity of the slide test, all positive slides and a random sample of some negative slides were reexamined by another experienced laboratory technician who was blind to the first slide-reader's diagnosis.

For the modified Ziehl–Neelsen method, a smear from the remaining sediment was stained with carbol fuchsin for 15 minutes and fixed with methanol for 2–3 minutes. The stain was decolorized with 1% acid alcohol for 15 seconds and counterstained with methylene blue for 30 seconds [11].

### 2.7. Data Processing and Analysis

The IBM SPSS statistical package (version 23) was used to enter and analyze the collected data [30]. The findings were presented using descriptive statistical methods (frequency and percentage). The data was summarized using frequency tables and a bar chart. Bivariant logistic regression was used to identify factors associated with endoparasites. The backward method was used. A reference variable was used to define the categorical variables, which was the last variable. An odds ratio and a *p* value were used to test the association. In the univariate analysis, factors having a *p* value < 0.2 were included in the multivariate analysis to control for the possible effect of confounders. Model fitness was checked using the Hosmer-Lemeshow test of goodness of fit before the actual logistic regression analysis. The adjusted odds ratio and *p* value *<* 0.05 were used to declare the final association.

### 2.8. Ethical Consideration

An ethical clearance letter (reference number: RCC1103/21) was obtained from the research ethics review committee of the College of Health Sciences, Debre Tabor University. Permission to conduct the study was sought from the respective hospital authorities. Informed written consent was obtained from the mothers/guardians of each child before enrolment in the study. Those mothers/guardians who were able to read and write signed the consent form themselves. Those who were unable to read and write provided their thumbprint after the information sheet, and consent form were read to them. The interview was not recorded on tape. Participants were informed that participation in the study was entirely voluntary and that they had the right to withdraw at any moment. Personal identities were not recorded on the questionnaire, and all data gathered through face-to-face interviews was kept with complete confidentiality. Besides, an explanation was given about the procedure of stool examination, which is noninvasive and causes no harm to the study participants, and the aim of the study result may benefit the study participants and the community as well. All the under-five children who were positive for any of the suspected intestinal parasites were linked to a pediatrician to be treated.

## 3. Results

### 3.1. Sociodemographic Characteristics of the Participants

In the present study, a total of 258 under-five children and their mothers/guardians were involved in the study, with a response rate of 100%. More than half of the respondents, 137 (53.10%), were females, and 159 (61.63%) were in the age group of 24 to 59 months. The majority of mothers/guardians' religions (240, 93.02%) were orthodox, and (225, 87.21%) were of Amhara ethnicity. One hundred forty-two (55.04%) children were from families having a monthly income of between 2000 and 3000 Ethiopian Birr (Table 1).

### 3.2. Health Information of the Participants

One hundred fifty-one (58.53%) of the mothers/guardians reported that they were frequently supervised by health professionals. One hundred thirty-four (51.94%) mothers/guardians reported having received health messages 1 week before the time of the survey, and the commonest source of information was government health workers, which accounted for 122 (91.04%). One hundred thirty-seven (53.10%) mothers/guardians reported that they exchanged health information within the family on a weekly basis (Table 2).

### 3.3. Characteristics of Food, Personal, and Environmental Hygiene

In this study, the majority (217, 84.11%) of participants had clean dining utensils. More than half (150, 58.14%) and 165 (63.95%) of mothers/guardians wash their hands after toileting and trim their children's nails when grown, respectively. Two hundred thirty-four (90.70%) of mothers/guardians had tap water as a source of drinking water, and 52 (20.16%) of mothers/guardians knew both contaminated food and water as a mode of transmission of intestinal parasites (Table 3).

### 3.4. Prevalence of Endoparasites

**Figure 1:**
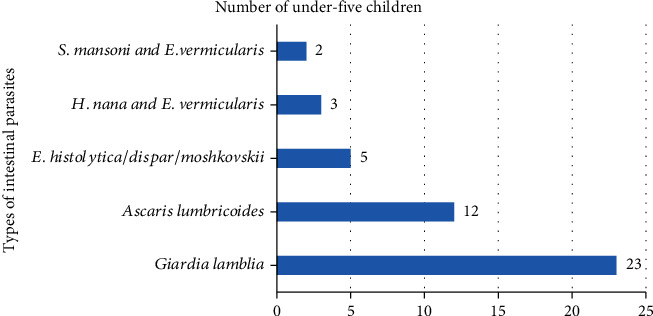
Types of intestinal parasites among under-five children attending at Debre Tabor comprehensive specialized hospital, Northwest Ethiopia, 2021 (*n* = 258).

The overall prevalence of one or more endoparasites among under-five children was 45 (17.44%). Among the identified intestinal parasites, the predominant one was *Giardia lamblia* 23 (51.11%), followed by *Ascaris lumbricoides* 12 (26.67%). The distribution of identified parasites among under-five children is described in Figure 1.

### 3.5. Risk Factors Associated with Endoparasites

Multivariate logistic regression analysis showed that health supervision, child food freshness, regular trimming of fingernails, and children's playground cleanliness were significantly associated with childhood endoparasites. However, there was no statistically significant association between the endoparasites and other variables (*p* value >0.05). The prevalence of childhood endoparasites was 2.52 times higher among households that had not been frequently supervised by health professionals (AOR = 2.52, 95%CI = [0.55, 1.50]). Children who were rarely fed a fresh meal were 4.61 (AOR = 4.61, 95%CI = [1.03, 4.12]) more likely to be infected by endoparasites than children fed a fresh meal. Those children whose nails were trimmed sometimes were 3.26 (AOR = 3.26, 95%CI = [1.91, 9.25]) more likely to be infected by endoparasites than children whose nails were cut always. Besides, children who did not have access to a clean playing ground were 2.67 times (AOR =2.67, 95% CI = [1.64, 6.01]) more likely to be infected by endoparasites than children who had access to a clean playing ground (Table 4).

## 4. Discussion

In underdeveloped nations, endoparasites are one of the primary causes of death among children [31]. To develop successful preventative and therapeutic approaches for child morbidity, it is critical to understand the distribution and extent of endoparasites in a specific population among susceptible groups such as children. The current study looked at the prevalence of endoparasites and associated risk factors among under-five children who visited the Debre Tabor comprehensive specialty hospital's pediatric ward.

Recently, the overall prevalence of one or more endoparasites among under-five children was 45 (17.44%). When the prevalence of endoparasites is less than 20%, it is considered the lowest in Ethiopia. According to the national classification of endoparasite prevalence, the finding of the current study has reached the lowest category. In addition, it is far from the short-term national target reduction of endoparasites of less than 1% in Ethiopia. So, it is impossible to meet the national target of eliminating parasites in 2020 [9]. The finding of this study was corroborated with what was found in Debre Birhan (17.4%) [5], Gondar (17.3%) [10], and Saudi Arabia (17.7%) [32]. This similarity could be due to the fact that studies used similar study designs and selected participants.

The current prevalence was greater than the surveys conducted in Portugal (7.8%) [33], Nigeria (13.7%) [31], and Dessie (15.5%) [11]. This may be due to the fact that 41.47% of households reported that they were not frequently supervised by health professionals in Debre Tabor. The prevalence was lower than the findings of previous studies conducted in different parts of Ethiopia, such as Woreta (18.7%) [12], Bahir Dar (20.4%) [13], Wolaita Sodo (21.2%) [14], Wonji Shoa Sugar Estate (24.3%) [15], Dembiya (25.4%) [16], Hawassa (26.6%) [17], Haro Dumal (38.5%) [18], Southern Ethiopia (41.9%) [19], Boricha (48.7%) [20], Hawassa Zuria (51.3%) [21], Senbete and Bete (52.3%) [6], and Wondo Genet (85.1%) [2]. This discrepancy could be due to the fact that the study subjects and data collection tools utilized in each study differ, which could have influenced the results. In our case, we were not only including children who were presenting with diarrheal diseases but also including children who did not have diarrheal disease complaints. But in Hawassa [17], the study subjects were under-five children who were presenting with diarrheal disease, which could increase the prevalence of the latter.

This study also showed low prevalence as compared with studies conducted in different parts of the world like Zambia (19.6%) [7], Sudan (24.9%) [34], Senegal (26.2%) [35], Uganda (26.5%) [36], Côte d'Ivoire (62.75%) [4], and Kenya (86%) [37]. The variation in prevalence could be due to the difference in geographical location, time of survey, varied characteristics of the study subjects, differences in sample size, implementation of different prevention and control measures, and socioeconomic status. However, the finding of our study is still higher according to the national safe environment strategy in the extension program in Ethiopia.

According to the prevalence rate of each parasite species, *Giardia lamblia* was the predominant prevailing parasite with a prevalence of 51.11%, followed by *Ascaris lumbricoides* (26.67%). This finding is comparable with the study conducted in Debre Birhan, Senbete and Bete, Wolaita Sodo, Nigeria, and Saudi Arabia [5, 6, 14, 31, 32], in which *Giardia lamblia* was most prevalent. Some studies indicated that the predominant parasite among children was *E. histolytica*/*E. dispar* [11, 13, 17]. This variation might be due to variations in study sample size and the involvement of different factors in the transmission of parasites. In the current study, double parasitic infections were detected in five children, which is similar to a study conducted in Debre Birhan five [5], whereas in Hawassa six [17], in Senbete and Bete towns 18 [6], and in Senegal 22 [35] children were infected with two parasites.

In this study, it was shown that health supervision, child food freshness, regular trimming of fingernails, and children's playground cleanliness were significantly associated with childhood endoparasites. The prevalence of endoparasites was higher among households that had not been frequently supervised by health professionals. Another study also reported the association of health supervision and endoparasites [16]. This fact can be justified by the fact that health supervision promotes health behaviors toward hygiene and sanitation practices. Health supervision increases knowledge and acceptability of interventions within the community. It also sustains integrated control of the infection.

Eating rarely fresh food was found to be a risk factor for endoparasites when compared to those who were fed always fresh food. This is because of the fact that storage of cooked food for a longer period gives rise to a proliferation of bacteria and other parasites. This result is supported by similar studies conducted in Ethiopia [18, 20]. Irregular trimming of children's fingernails was also significantly associated with endoparasites. This result is supported by similar studies conducted in Ethiopia [5, 12, 18]. This could be attributed to the removal of accumulated dirt containing eggs of parasites on fingernails during trimming [38].

Children who had an unclean playground were found to be at an increased rate of having endoparasites (AOR = 2.67, 95%CI = [1.64, 6.01]). This is due to the presence of microorganisms and eggs of parasites on dirty surfaces. This is supported by a study which concluded that inadequate sanitation and hygiene behavior are associated with soil-transmitted helminths and intestinal protozoa infections [39].

In the present study, there was no significant association between gender and endoparasites. Similarly, other research studies in Ethiopia revealed the prevalence of endoparasites did not show any association with gender [5, 15, 17]. In contrast to this study finding, a study in Kenya showed an association between endoparasites and gender (*p* value =0.045) [40].

Mothers/guardians who had a low level of education had a higher risk of their children acquiring endoparasites than other mothers/guardians who had a higher education [5, 32]. But the current study did not show an association between endoparasites and mothers/guardians' education status. This is in line with the study done in Dessie [11]. In contrast to this study's finding, other studies showed an association between endoparasites and drinking water from a river source and age [5, 15, 17].

Although these findings are limited to one hospital, because of the extensive range of health services provided to children in that hospital, they may represent the population of the area. All of this research suggests that intervention measures to reduce the spread of endoparasites in this context should be implemented effectively. Furthermore, for efficient control of endoparasites, this study highlights the importance of focused health education, health awareness, and cleanliness practices.

## 5. Limitations of the Study

There were only a few limitations to this study. First, because of the cross-sectional design, any potential temporal relationships were difficult to analyze. Second, some study variables may be subject to recall bias. Third, children are only recruited within two months, and there may be seasonal fluctuations in the prevalence of endoparasites in the study area. Finally, other factors that could influence the prevalence of endoparasites, such as family size, the number of rooms used to sleep, and contact with animals, were not addressed.

## 6. Conclusion

Endoparasites, primarily *Giardia lamblia* and *Ascaris lumbricoides*, were found to be relatively prevalent among under-five children in the study area. Despite the health intervention coverage of Ethiopians by the Ministry of Health, the present study demonstrated a higher prevalence of endoparasites among under-five children, which needs holistic and integrated efforts to control endoparasites. Health supervision, child food freshness, regular trimming of fingernails, and children's playground cleanliness were significantly associated with endoparasites. Thus, strengthening health education about food, personal, and environmental hygiene for both children and their mothers/guardians is crucial. Besides, improving mothers/guardians' awareness about the mode of intestinal parasite transmission and prevention methods is necessary.

## Figures and Tables

**Table 1:** Sociodemographic characteristics of under-five children and their caregivers in Debre Tabor comprehensive specialized hospital, Northwest Ethiopia, 2021 (n =258).

Variables	Frequency	Percent (%)
Gender		
Male	121	46.90
Female	137	53.10
Age (in months)		
6-11	46	17.83
12*–*23	53	20.54
24*–*59	159	61.63
Family residence		
Urban	108	41.86
Rural	150	58.14
Religion of mother/guardian		
Orthodox	240	93.02
Muslim	8	3.10
Protestant	10	3.88
Ethnicity of mother/guardian		
Amhara	225	87.21
Tigre	33	12.79
Occupation of mother/guardian		
Civil servant	98	37.98
Housewife	74	28.68
Merchant	25	9.69
Farmer	61	23.65
Mother/guardian's educational status		
Unable to read and write	70	27.13
Able to read and write	65	25.19
Grade 1–8	23	8.92
Grade 9–12	28	10.85
Certified and above	72	27.91
Monthly family income		
<2000 Et Birr	67	25.97
2000*–*3000 Et Birr	142	55.04
>3000 Et Birr	49	18.99

**Table 2:** Baseline Health care related information of children caregivers respondents in Debre Tabor comprehensive specialized hospital, Northwest Ethiopia, 2021 (*n* = 258).

Variables	Frequency	Percent
A health professional frequently visits the household		
Yes	151	58.53
No	107	41.47
Mother/guardian received health messages last week prior to the survey		
Yes	134	51.94
No	124	48.06
Source of health messages		
Government health workers	122	91.04
Church leaders	6	4.48
Radio	3	2.24
Community discussion	3	2.24
Mothers/guardians exchange health information within the family on a regular basis		
Yes	137	53.10
No	121	46.90

**Table 3:** Characteristics of food, personal, and environmental hygiene of participants at Debre Tabor comprehensive specialized hospital, Northwest Ethiopia, 2021 (*n* = 258).

Variables	Frequency	Percent (%)
Are dinning utensils clean?		
Yes	217	84.11
No	41	15.89
Do you wash your hands after using the toilet before touching your child?		
Always	150	58.14
Sometimes	108	41.86
Does your child eat raw or unwashed vegetables and fruits?		
Always	173	67.05
Sometimes	76	29.46
Never	9	3.49
Your child meal		
Always fresh	134	51.94
Sometimes fresh	101	39.15
Rarely fresh	23	8.91
Do you trim your child's nails when they grow?		
Always	165	63.95
Sometimes	93	36.05
Does your child take food other than breast milk before the age of six months?		
Yes	132	51.16
No	126	48.84
Your child's playing ground		
Not clean	162	62.79
Clean	96	37.21
What is your source of drinking water?		
Tap water	234	90.70
Stream water	24	9.30
The type of toilet you have		
Open defecation	8	3.10
Public	29	11.24
Private	221	85.66
Walking on bare foot		
Yes	41	15.89
No	217	84.11
Knowledge of the mode of transmission		
Contaminated food	108	41.86
Contaminated water	98	37.98
Both	52	20.16

**Table 4:** Bi-variable and multi-variable logistic regression to determined risk factors for acquiring endoparasites for under-five children attending in Debre Tabor comprehensive specialized hospital, Northwest Ethiopia, 2021 (*n* = 258).

Variables	Parasite infection		COR (95% CI)	AOR (95% CI)
	Positive/yes	Negative/no		
Family residence				
Urban	17	91	1	1
Rural	28	122	1.98 (0.97–5.83)∗	2.01 (0.62–4.26)
Occupation of mother/guardian				
Civil servant	4	94	1	1
Housewife	20	54	2.92 (0.93–4.80)∗	2.81 (0.25–8.90)
Merchant	9	16	0.69 (0.21–1.94)	0.42 (0.16–1.68)
Farmer	12	49	0.84 (0.24–2.63)	1.22 (0.29–4.18)
Mother/guardian's educational status				
Unable to read and write	19	51	1	1
Able to read and write	12	53	3.35 (0.52–3.98)∗	2.81 (0.43–5.02)
Grades 1–8	6	17	0.42 (0.19–1.31)	0.51 (0.50–1.37)
Grades 9–12	4	24	0.86 (0.57–3.32)	1.80 (0.49–4.34)
Certified and above	4	68	1.22 (0.38–4.42)	0.58 (0.19–16.46)
A health professional frequently visits the household				
Yes	16	135	1	1
No	29	78	2.47 (0.31–1.53)∗	2.52 (0.55–1.50)^**†**^
Does your child eat raw or unwashed vegetables and fruits?				
Always	32	141	3.14 (1.59–10.72)∗	1.87 (0.83–6.71)
Sometimes	10	66	1.95 (1.24–4.89)	1.04 (0.76–2.56)
Never	3	6	1	1
Your child meal				
Always fresh	4	130	1	1
Sometimes fresh	29	72	1.18 (1.57–7.36)	1.34 (1.45–6.59)
Rarely fresh	12	11	4.47 (0.73–5.12)∗	4.61 (1.03–4.12)^**†**^
Do you trim your child's nails when they grow?				
Always	6	159	1	1
Sometimes	39	54	3.46 (1.42–10.06)∗	3.26 (1.91–9.25)^**†**^
Your child's playing ground				
Not clean	26	136	2.96 (2.11–3.87)∗	2.67 (1.64–6.01)^**†**^
Clean	19	77	1	1
What is your source of drinking water?				
Tap water	17	217	1	1
Stream water	21	3	1.42 (1.48–3.56)∗	1.61 (0.92–4.39)
The type of toilet you have				
Open defecation	6	2	1.63 (0.47–0.94)∗	1.40 (2.47–9.16)
Public	15	14	0.28 (0.18–0.65)	1.74 (0.98–9.08)
Private	24	197	1	1

Key: COR = crude odds ratio; AOR = adjusted odds ratio; CI = confidence interval; 1 = reference categories; ∗ = indicates significant association (*p* value <0.2); ^**†**^ = indicates significant association (*p* value <0.05). Goodness of fit test: the Hosmer-Lemeshow *χ*^2^ (8df) = 7.81; *p* = 0.46.

## Data Availability

The datasets for analyzed the current study are available from the corresponding author upon reasonable request of him.

## Supplementary Materials

Supplementary Materials The supplementary material consisted of four parts. The first part embraced questions about the sociodemographic profile of respondents, such as gender and age of child, family residence, religion, ethnicity, occupation, mother/guardian's educational status, and family income. The second was to inquire about health information. The third was characteristics of food, personal, and environmental hygiene. The fourth part was about the types of intestinal parasites with **diagnostic techniques.**
