# Supplementary material for: Prevalence and Associated Risk Factors of Endoparasites among Under-Five Children in Debre Tabor Comprehensive Specialized Hospital, Debre Tabor, Northwest Ethiopia: A Cross-Sectional Study
Source: J Parasitol Res. 2022 Apr 30;2022:6917355. doi: 10.1155/2022/6917355 (PMC9078827; doi:10.1155/2022/6917355)
Supplement: Supplementary Materials — The supplementary material consisted of four parts. The first part embraced questions about the sociodemographic profile of respondents, such as gender and age of child, family residence, religion, ethnicity, occupation, mother/guardian's educational status, and family income. The second was to inquire about health information. The third was characteristics of food, personal, and environmental hygiene. The fourth part was about the types of intestinal parasites with diagnostic techniques. [file 6917355.f1.docx]

**Data collection tool (questionnaire)**

**Title of the study:** Prevalence and associated risk factors of intestinal parasitic infections among under-five children in Debre Tabor comprehensive specialized hospital, Debre Tabor, Northwest Ethiopia: A cross-sectional study

1. **Sociodemographic characteristics of the participants**
2. Gender
3. Male
4. Female
5. Age (in months) __________
6. Family residence
7. Urban
8. Rural
9. Religion of mother/guardian
10. Orthodox
11. Muslim
12. Protestant
13. Ethnicity of mother/guardian
14. Amhara
15. Tigrie
16. Occupation of mother/guardian
17. Civil servant
18. Housewife
19. Merchant
20. Farmer
21. Mother/guardian's educational status
22. Unable to read and write
23. Able to read and write
24. Grade 1–8
25. Grade 9–12
26. Certificate and above
27. Monthly family income
28. < 2000 Et Birr
29. 2000–3000 Et Birr
30. > 3000 Et Birr
31. **Health information of the participants**
32. A health professional frequently visits the household
33. Yes
34. No
35. Mother/guardian received health messages last week prior to the survey
36. Yes
37. No
38. Source of health messages
39. Government health workers
40. Church leaders
41. Radio
42. Community discussion
43. Mothers/guardians exchange health information within the family on a regular basis
44. Yes
45. No
46. **Characteristics of food, personal, and environmental hygiene**
47. Are dinning utensils clean?
48. Yes
49. No
50. Do you wash your hands after using the toilet before touching your child?
51. Always
52. Sometimes
53. Does your child eat raw or unwashed vegetables and fruits?
54. Always
55. Sometimes
56. Never
57. Your child meal
58. Always fresh
59. Sometimes fresh
60. Rarely fresh
61. Do you trim your child’s nails when they grow?
62. Always
63. Sometimes
64. Does your child take food other than breast milk before the age of six months?
65. Yes
66. No
67. Your child's playing ground
68. Not clean
69. Clean
70. What is your source of drinking water?
71. Tap water
72. Stream water
73. The type of toilet you have
74. Open defecation
75. Public
76. Private
77. Walking on bare foot
78. Yes
79. No
80. Knowledge of the mode of transmission
81. Contaminated food
82. Contaminated water
83. Both
84. **Types of intestinal parasites**

| **S.No** | **Name of parasite species** | **Diagnostic techniques** | **Remark** |
| --- | --- | --- | --- |
|  |  |  |  |
|  |  |  |  |
|  |  |  |  |
|  |  |  |  |
|  |  |  |  |
